# Laryngopharyngeal reflux as a potential cause of Eustachian tube dysfunction in patients with otitis media with effusion

**DOI:** 10.3389/fneur.2022.1024743

**Published:** 2022-11-03

**Authors:** Zhen Zhen, Tingting Zhao, Quangui Wang, Junbo Zhang, Zhen Zhong

**Affiliations:** ^1^Department of Otolaryngology, Head and Neck Surgery, Peking University First Hospital, Beijing, China; ^2^Department of Head and Neck Surgery, National Cancer Center/National Clinical Research Center for Cancer/Cancer Hospital and Shenzhen Hospital, Chinese Academy of Medical Sciences and Peking Union Medical College, Shenzhen, China

**Keywords:** laryngopharyngeal reflux, Eustachian tube, otitis media with effusion, tubomanometry (TMM), Reflux Symptom Index (RSI)

## Abstract

**Objective:**

To explore the association between laryngopharyngeal reflux disease (LPRD)-related symptoms and the Eustachian tube (ET) function in adult patients with otitis media with effusion (OME).

**Materials and methods:**

A total of 105 adult patients with OME were retrospectively studied. All these patients had undergone tubomanometry (TMM) test for the affected ears before treatments. The LPRD-related symptoms were all assessed by the Reflux Symptom Index (RSI) scale.

**Results:**

Among the 105 included patients, the numbers of subjects with only one and both two ears affected were 65 (57.1%) and 40 (42.9%), respectively. Therefore, a total of 145 affected ears were studied. For these affected ears, a linear regression analysis that included sex, age, BMI, smoking history, drinking history, RSI value, and the condition of the contralateral ear suggested that only RSI value was significantly associated with TMM value (*P* < 0.001), with the correlation coefficient of −0.112. Among the 9 symptoms in RSI scale, affected ears with the following symptoms (vs. affected ears without) showed significantly lower TMM values: excess throat mucus or postnasal drip, difficulty swallowing food, liquids, or pills, and sensations of something stuck in your throat or a lump in your throat (all *P* < 0.05).

**Conclusion:**

LPRD may disrupt ET function in adult OME patients. A higher RSI score is independently predictive for a bad ET patency in such patients and is indicative for an additional anti-reflux therapy.

## Introduction

Otitis media with effusion (OME) is a non-suppurative disease characterized by the presence of fluid in the middle ear cavity without acute infection. Its main symptoms include hearing loss, ear fullness, earache, and tinnitus. Although there is a certain chance of self-healing, there are still a great number of such patients who need treatments. When treated inappropriately, this disease may cause long-term complications such as adhesive otitis media, cholesteatoma, tympanosclerosis, and ossicular necrosis (1).

Currently, there are not standard criteria for treating adult OME. Conservative treatments including medication or the combination of medication and Eustachian tube (ET) auto-inflation are usually preferred for newly diagnosed patients (2, 3), with advantages of non-invasiveness and good compliance. However, conservative treatments are usually less effective for those with bad ET patency (4, 5). Therefore, exploring factors related to ET function are important for a precise treatment for this disease.

Laryngopharyngeal reflux disease (LPRD) refers to an inflammatory condition of the upper aerodigestive tract tissues related to gastric or duodenal content reflux (6, 7). This disease could be associated with laryngological, rhinological, and ontological conditions (8–10). Animal experiments have confirmed the role of such reflux in disrupting the ET patency through decreased ciliary clearance, mucosal hyperemia, and edema (11). However, few clinical researches are available with respect to the effects of LPRD on ET function.

The present study aimed to investigate the correlation between LPRD and ET function assessed by tubomanometry (TMM) in adult OME patients. The occurrence and severity of LPRD were assessed by Reflux Symptom Index (RSI), a widely used subjective scale proposed by Belafsky et al. (12). These results may be important reference for the indication of anti-reflux therapy in OME patients.

## Materials and methods

### Study population

Between Jan 2019 and Jan 2020, a total of 105 newly diagnosed OME patients in our database who met the following criteria were retrospectively studied: (1) age ≥18 years; (2) typical primary complaints included ear fullness and (or) hearing loss of more than 2 weeks; (3) conductive hearing loss on pure tone test and type B or C results from the tympanometry test; (4) an intact tympanic membrane and no nasopharyngeal neoplasms or adenoidal hyperplasia according to endoscopy examination; (5) no upper respiratory allergy or infection during the course of treatments; and (6) complete RSI assessment data. The baseline information of all studied patients including age, sex, body mass index (BMI), smoking history, drinking history, and RSI value are shown in Table 1. This study protocol was approved by the ethics committee of Peking University First Hospital.

**Table 1:** The baseline information of the 105 newly diagnosed OME patients.

	**Numbers/**	**Percentage**
	**Mean ±SD**	**(%)/range**
Age (years)	49.9 ± 15.3	18–87
**Sex**		
Male	47	44.8
Female	58	55.2
BMI (kg/m^2^)	24.4 ± 3.6	14.9–33.2
**Smoking history**		
Yes	21	20
No	84	80
**Drinking history**		
Yes	11	10.5
No	94	89.5
RSI value	5.1 ± 5.8	0-30

### TMM

The TMM (Spiggle and Theis, Overath, Germany) was performed for all affected ears in our patients as described previously (13). The patients took a sitting position with the pressure receptor probe set in the ear canal. After the patients held a small amount of water in the mouth, a nasal adapter was set into both nostrils. The patients were then told to tightly close the teeth and swallow. The pressure receptor probe records the pressure changes transmitted through the movements of the tympanic membrane. The pressure changed in the nasopharynx and external auditory canal also could be recorded; the data were saved on a computer, and figures measure the pressure changes in the nasopharynx and ear canal in millibars (mbar) against time in seconds. 30, 40, and 50 mbar were used as three initial nasal cavity pressures in the test. The functional state of the ET was evaluated using the opening latency index (R value) by R = (P1 – C1)/(C2 – C1). P1 is the start of the movement of the eardrum after pressure application, C1 is the start of the pressure increase in the nasopharynx, and C2 is the maximum pressure increase in the nasopharynx. P1 – C1 represents the latency of tubal opening, and C2 – C1 represents the time of increased pressure in the nasopharynx. The R value quantifies the ET patency: an R value ≤1 indicates satisfactory ET function and is weighted as two points, indicating that the tube opening occurs before C2; an *R* value >1 suggests a delayed opening or restricted ET function and is weighed as 1 point, indicating that the tube opening occurs after C2; while a negative or non-measurable R value indicates that the ET has not opened or is occluded and is weighted as 0 point. The TMM values are calculated as the sum of all points at 30, 40, and 50 mbar; hence, the theoretical range of TMM should be 0–6 (13).

### RSI assessment

RSI is a 9-item patient questionnaire scoring system used to assess severity of reflux symptoms. Each item of RSI was scored from 0 (no symptom) to 5 (the most serious symptom), and the total score can be between 0 and 45 points. The questionnaire was distributed on site by one designated researcher to all the OME patients and was collected after they had completed it.

### Statistical analysis

The statistical analysis in current study was all performed with SPSS software version 20.0 (IBM, Armonk, NY). Continuous variables were all displayed as mean ± standard deviation. The unpaired Student *t*-test and one-way ANOVA were used to compare continuous variables between different groups. The linear regression analysis was used to identify significant associations. For all analyses, a *P*-value < 0.05 was considered to be statistically significant.

## Results

Among the 105 included patients, the numbers of subjects with only one and both two ears affected were 65 (57.1%) and 40 (42.9%), respectively. Therefore, a total of 145 affected ears were studied. The overall TMM value of these ears was 3.9 ± 2.2, with a range of 0–6. The RSI score according to different TMM values was shown in Table 2, suggesting a decrease tend of RSI with an increase of TMM value (*P* = 0.007).

**Table 2:** The TMM value distribution of all 145 affected ears and corresponding RSI values.

**TMM**	**No. of ears**	**RSI value**	***P*-value**
0	20	7.1 ± 7.3	0.007
1	8	11.4 ± 9.5	
2	16	6.1 ± 5.9	
3	15	5.2 ± 4.2	
4	12	4.8 ± 4.7	
5	19	5.0 ± 5.6	
6	55	3.4 ± 4.3	

A linear regression analysis including sex, age, BMI, smoking history, drinking history, RSI value, and the condition of the contralateral ear suggested that only RSI value was significantly associated with TMM value (*P* < 0.001). The coefficient of this correlation was −0.112 (SE 0.03) and the *R*^2^ of this model was 0.087 (adjusted *R*^2^ 0.081, SE of the estimate 2.123).

The TMM value was also compared according to different items of RSI scale. As shown in Table 3, affected ears with the following symptoms (vs. affected ears without) showed significantly lower TMM values: excess throat mucus or postnasal drip, difficulty swallowing food, liquids, or pills, and sensations of something stuck in your throat or a lump in your throat (all *P* < 0.05).

**Table 3:** The comparison of TMM value between ears with different RSI symptoms.

**Items of RSI scale**	**No. of ears**	**TMM value**	***P***
Hoarseness or problem with your voice			0.236
Yes	32	3.4 ± 2.2	
No	113	4.0 ± 2.2	
Clearing your throat			0.066
Yes	59	3.4 ± 2.3	
No	86	4.1 ± 2.2	
Excess throat mucus or postnasal drip			0.015
Yes	71	3.4 ± 2.3	
No	74	4.3 ± 2.1	
Difficulty swallowing food, liquids, or pills			0.006
Yes	12	2.2 ± 2.1	
No	133	4.0 ± 2.2	
Coughing after you ate or after lying down			0.109
Yes	27	3.2 ± 2.5	
No	118	4.0 ± 2.1	
Breathing difficulties or choking episodes			0.367
Yes	22	3.5 ± 2.2	
No	123	3.9 ± 2.2	
Troublesome or annoying cough			0.061
Yes	36	3.3 ± 2.3	
No	109	4.1 ± 2.1	
Sensations of something stuck in your throat or a lump in your throat			0.030
Yes	62	3.4 ± 2.1	
No	83	4.2 ± 2.2	
Heart burn, chest pain, indigestion, or stomach acid coming up			0.175
Yes	39	3.4 ± 2.3	
No	106	4.0 ± 2.2	

## Discussion

ET is a potential ventilation passage connecting the middle ear cavity to the nasopharynx with the primary function of adjusting middle ear pressure (14). ET dysfunction plays an important role in the pathogenesis and progression of OME (15, 16). Currently, there have been several studies that had explored the relationship between OME and LPRD (17, 18), with some ones had directly detected pepsin/pepsinogen in middle ear secretions (19–21). However, the effect of LPRD on ET function in OME patients is not clear. This is mainly due to the lack of accurate methods in evaluating the ET patency in the past. The TMM method introduced by Schroder et al. is a relatively new semi-objective method for evaluating ET patency (13). Using this method, we investigated the potential effect of LPRD on ET function among OME patients.

The most important finding of present study was that a high RSI score was independently predictive for a low TMM value, suggesting an important role of LPRD in damaging ET function of OME patients. This not only further proved that LPRD can be involved in the occurrence and progression of OME by affecting ET function, but also suggested that the simple RSI score could be used as a reference for anti-reflux therapy in adult OME patients. As the ET dysfunction evaluated by TMM method had been proved to be associated with ineffective conservative treatments (4, 5), the clinical research conducted by Pang et al. had proved the potential advantages of acid-suppressive drugs in OME patients with abnormal RSI results (22). Our results further suggested that the mechanism may be attributed to the prevention of LPRD in disrupting ET patency. Therefore, an additional anti-reflux therapy should be suggested for OME patients with abnormal RSI scores.

In current study, we also explored what symptoms included in RSI scale are more likely to be associated with a lower TMM value and found them to be excess throat mucus or postnasal drip, difficulty swallowing food, liquids, or pills, and sensations of something stuck in your throat or a lump in your throat. As a result, OME patients with such reflux symptoms may benefit more from an additional anti-reflux therapy.

The study had some limitations that need to be addressed. First, there was no further confirmatory research that could explore the effect of anti-reflux therapies in improving ET function. This is important and has been added into our future research plans. Second, the RSI scale is not an objective method in evaluating LPRD and its accuracy can be easily affected by some subjective factors. Some other objective evaluations of LPRD which were not collected may be more useful in predicting TMM values, such as salivary pepsin value or pH test results. However, there were some studies that had proved the close relationship between RSI with the diagnostic gold standards of LPRD (dynamic multi-probe esophageal impedance and PH value monitoring) (23, 24). At last, we did not detect the mechanism by which LPRD affects ET function. We hypothesized that local fluid mechanic and micro-environment changes brought by reflux may be the main reasons, which need further researches.

## Conclusions

In summary, the current study suggested that LPRD may disrupt ET function in OME patients. A higher RSI score is independently predictive for a bad ET patency in such patients and is indicative for an additional anti-reflux therapy.

## Data availability statement

The raw data supporting the conclusions of this article will be made available by the authors, without undue reservation.

## Ethics statement

The studies involving human participants were reviewed and approved by the Ethics Committee of Peking University First Hospital. Written informed consent for participation was not required for this study in accordance with the national legislation and the institutional requirements.

## Author contributions

JZ and ZZho designed the study and revised the manuscript. ZZhe, TZ, and QW participated in the material preparation, data collection, data analysis, and drafting of the manuscript. All authors commented on previous versions of the manuscript, read, and approved the final manuscript.

## Funding

This study was supported by Natural Science Foundation of Beijing Municipality (7204315), Youth clinical research project of Peking University First Hospital (2019CR30), and Capital Foundation of Medical Development (2022-2-4078).

## Conflict of interest

The authors declare that the research was conducted in the absence of any commercial or financial relationships that could be construed as a potential conflict of interest.

## Publisher's note

All claims expressed in this article are solely those of the authors and do not necessarily represent those of their affiliated organizations, or those of the publisher, the editors and the reviewers. Any product that may be evaluated in this article, or claim that may be made by its manufacturer, is not guaranteed or endorsed by the publisher.
